# Engineering Musculoskeletal Grafts for Multi-Tissue Unit Repair: Lessons From Developmental Biology and Wound Healing

**DOI:** 10.3389/fphys.2021.691954

**Published:** 2021-08-24

**Authors:** Xu Zhang, Dan Wang, King-Lun Kingston Mak, Rocky S. Tuan, Dai Fei Elmer Ker

**Affiliations:** ^1^Institute for Tissue Engineering and Regenerative Medicine, The Chinese University of Hong Kong, Shatin, China; ^2^Faculty of Medicine, School of Biomedical Sciences, The Chinese University of Hong Kong, Shatin, China; ^3^Department of Orthopaedics and Traumatology, Faculty of Medicine, The Chinese University of Hong Kong, Shatin, China; ^4^Key Laboratory for Regenerative Medicine, Ministry of Education, School of Biomedical Sciences, The Chinese University of Hong Kong, Shatin, China; ^5^Bioland Laboratory (Guangzhou Regenerative Medicine and Health-Guangdong Laboratory), Guangzhou, China

**Keywords:** bone, tendon, muscle, multi-tissue units, musculoskeletal developmental biology, wound healing, musculoskeletal tissue engineering, inflammation

## Abstract

In the musculoskeletal system, bone, tendon, and skeletal muscle integrate and act coordinately as a single multi-tissue unit to facilitate body movement. The development, integration, and maturation of these essential components and their response to injury are vital for conferring efficient locomotion. The highly integrated nature of these components is evident under disease conditions, where rotator cuff tears at the bone-tendon interface have been reported to be associated with distal pathological alterations such as skeletal muscle degeneration and bone loss. To successfully treat musculoskeletal injuries and diseases, it is important to gain deep understanding of the development, integration and maturation of these musculoskeletal tissues along with their interfaces as well as the impact of inflammation on musculoskeletal healing and graft integration. This review highlights the current knowledge of developmental biology and wound healing in the bone-tendon-muscle multi-tissue unit and perspectives of what can be learnt from these biological and pathological processes within the context of musculoskeletal tissue engineering and regenerative medicine. Integrating these knowledge and perspectives can serve as guiding principles to inform the development and engineering of musculoskeletal grafts and other tissue engineering strategies to address challenging musculoskeletal injuries and diseases.

## Introduction

Bone, tendon, and skeletal muscle are essential components of the musculoskeletal system whose development, integration, and response to injury are vital for conferring efficient body movement. Specifically, joint motion occurs as a result of bone, tendon, and skeletal muscle coordinately acting as a single multi-tissue unit, muscle-generated contractile force is transmitted to compliant tendons, which efficiently stores and subsequently releases elastic strain energy to result in bone movement. During development, these tissues are specified in a concerted manner, resulting in a highly integrated unit that further matures due to post-natal mechanical loading. This highly integrated nature is evident when injury is sustained. For example, in rotator cuff tears, although the bone-tendon interface or enthesis is often the site of injury, distal pathological alterations such as skeletal muscle degeneration in the form of fibrosis and fatty degeneration (Gilbert et al., 2018) as well as bone loss (Galatz et al., 2005) have been reported. Inflammation is a crucial factor that determines the outcome of tissue healing as well as the response to medical devices such as grafts. As such, knowledge gleaned from developmental biology and wound healing can serve as guiding principles to inform the development and engineering of musculoskeletal grafts.

Musculoskeletal tissue engineering aims to apply combinations of cells, signaling molecules including growth factors and inflammation-modulating factors, and biomaterials to generate mechanically-robust and/or bioactive grafts/scaffolds for treatment of injured or diseased tissues. Within the context of this review, we define a biomaterial as the base material or substance from which a scaffold or graft is fabricated. This means that a scaffold or graft is the engineered form of a biomaterial with the term “scaffold” being used in pre-clinical studies and “graft” being used in clinical settings. To appropriately utilize musculoskeletal development and wound healing knowledge and concepts, it is vital to recognize that the bone-tendon-muscle unit is a single functioning entity for locomotion (Ker et al., 2011a,b; Wang et al., 2021b). Therefore, understanding how these musculoskeletal tissues along with their interfaces develop, integrate, and mature (section “Brief Overview of Bone, Tendon, Muscle Development, Integration, and Maturation”) as well as the impact of inflammation on musculoskeletal healing and graft integration (section “Musculoskeletal Tissue Healing and Graft Integration”) is crucial. In addition, we present our perspective of what can be learnt from these biological processes within the context of musculoskeletal tissue engineering and regenerative medicine (section “Perspective: What Can We Learn From Developmental Biology and Wound Healing for Musculoskeletal Tissue Engineering”) as well as potential treatment strategies (section “Outlook”). Integrating such knowledge will facilitate the development of rational and informed tissue engineering strategies for addressing challenging musculoskeletal injuries and diseases.

## Brief Overview of Bone, Tendon, Muscle Development, Integration, and Maturation

### Musculoskeletal Tissue Development and Integration

**Figure 1:**
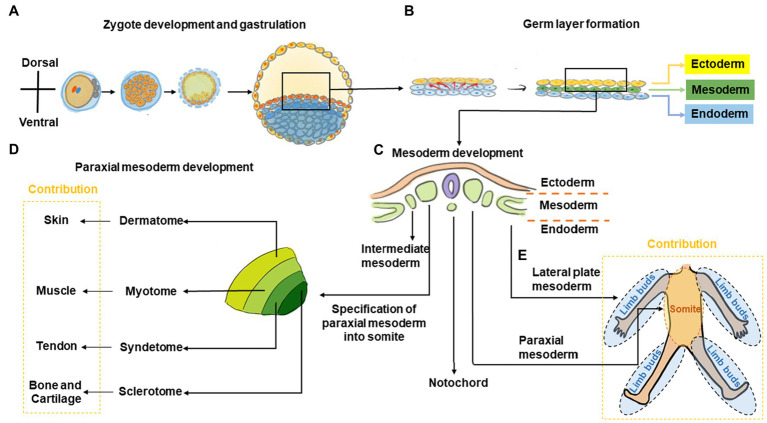
Graphic overview of musculoskeletal development in the trunk and limbs. **(A)** The fertilized zygote transitions through several embryonic structures before forming the blastula, which contains the two-layered embryonic or germ disk consisting of epiblast and hypoblast. **(B)** As development proceeds, a tri-laminar gastrula structure consisting of ectoderm, mesoderm, and endoderm layers is formed. **(C)** Further during gastrulation, the mesoderm subdivides into the paraxial, intermediate, and lateral plate mesoderm. **(D)** Subsequently, somites are formed from paraxial mesoderm. The somite further subdivides into the sclerotome, syndetome, myotome, and dermatome regions, which contribute toward cartilage and bone, tendon, muscle, and skin elements, respectively. **(E)** The lateral plate mesoderm gives rise to the limb bud, which forms the cartilage/bone and tendon elements of the appendicular skeleton, while migrating myoblasts from the myotome contribute toward muscle elements.

During embryonic development, the mesoderm is the prime contributor to musculoskeletal formation. The paraxial and lateral plate mesoderm, contribute toward somite and limb bud formation, respectively, which in turn are responsible for musculoskeletal formation in the trunk (primarily somitic in origin) and appendicular skeleton (primarily limb bud in origin; Figure 1; Cserjesi et al., 1995; Schweitzer et al., 2001; Wolpert et al., 2002; Mitchell and Sharma, 2005; Thomopoulos et al., 2010; Berendsen and Olsen, 2015; Endo, 2015; Jensen et al., 2018). The details for embryonic development of musculoskeletal tissue are comprehensively described in other excellent book chapters and reviews (Walker, 1991; Wolpert et al., 2002; Mitchell and Sharma, 2005; Schweitzer et al., 2010; Huang, 2017).

During trunk musculoskeletal development, somites are initially comprised of sclerotome and dermomyotome regions only but subsequently subdivide into the sclerotome, syndetome, myotome, and dermatome, which contribute toward bone, tendon, muscle, and skin elements, respectively (Cserjesi et al., 1995; Schweitzer et al., 2001; Wolpert et al., 2002; Mitchell and Sharma, 2005; Thomopoulos et al., 2010; Berendsen and Olsen, 2015; Endo, 2015; Jensen et al., 2018). Patterning signals including Sonic hedgehog (SHH; Fan and Tessier-Lavigne, 1994; Bumcrot and McMahon, 1995; Musumeci et al., 2015), Noggin (Berendsen and Olsen, 2015; Musumeci et al., 2015), Wingless/Integrated (Wnt), Neutrophin, and Fibroblast growth factor (FGF) as well as transcription factors including paraxis, paired box protein (PAX)-1, and PAX-9 are crucial to somite development (Love and Tuan, 1993; Smith and Tuan, 1995; Barnes et al., 1996a,b, 1997; LeClair et al., 1999; Alexander and Tuan, 2010). Within the sclerotome, cells form mesenchymal condensations that contribute to skeletal formation indirectly (*via* cartilaginous endochondral bone formation) or directly (*via* intramembranous bone formation; Berendsen and Olsen, 2015). Disrupting *Pax1* expression using antisense oligonucleotides or valproic acid disrupts sclerotomal differentiation to cause skeletal defects (Love and Tuan, 1993; Smith and Tuan, 1995; Barnes et al., 1996a,b, 1997; LeClair et al., 1999; Alexander and Tuan, 2010). As somite development progresses, a group of cells migrate away from the dorso-lateral edges of the dermomyotome toward the original boundary of the sclerotome and dermomyotome (Musumeci et al., 2015). This action segregates the dermomyotome into myotome and dermatome regions, and cells within this region undergo rapid muscle differentiation (Musumeci et al., 2015). Concurrently, muscle progenitor cells also undergo long-range migration to the limb buds, where they contribute toward appendicular muscle formation (Cossu et al., 1996; Musumeci et al., 2015). As the somite matures, a new domain termed the syndetome forms at the interface of sclerotome and myotome. Within the syndetome, tendon specification is initiated with the expression of scleraxis (*Scx*), a basic helix–loop–helix transcription factor that marks a tendon/ligament progenitor population and is subsequently present in terminally-differentiated tendon/ligament cells (Cserjesi et al., 1995; Brent et al., 2003). SCX is also known to upregulate tenomodulin (TNMD), a transmembrane protein found in tenocytes (Shukunami et al., 2006) as well as collagen type I, a main component of the tendon extracellular matrix (ECM; Lejard et al., 2007). Interactions among the abutting musculoskeletal compartments are crucial for further tissue development with transforming growth factor (TGF) and FGF signals from these regions regulating *Scx* expression and musculoskeletal integration (Brent and Tabin, 2004; Brent et al., 2005; Pryce et al., 2009). For example, mouse embryos with null mutations in Myogenic factor-5 (*Myf5*) and Myogenic differentiation 1 (*MyoD*) are devoid of skeletal muscle and their absence results in disruption of tendon development (Brent et al., 2005).

During development of the appendicular musculature and skeleton, the lateral plate mesoderm forms the limb bud, which contributes to bone and tendon elements, while migrating muscle cells originating from the myotome contribute to muscle elements (Cserjesi et al., 1995; Schweitzer et al., 2001; Wolpert et al., 2002; Mitchell and Sharma, 2005; Thomopoulos et al., 2010; Berendsen and Olsen, 2015; Endo, 2015; Jensen et al., 2018). Homeobox (*Hox*) transcription factor genes are important to this process and contribute toward patterning in the developing limb to define the stylopod (*Hox10*), zeugopod (*Hox11*), and autopod (*Hox13*) structures, which eventually give rise to the humerus/femur, radius and ulna/tibia and fibula, and hand/foot bones, respectively (Pineault and Wellik, 2014). Similar to its role in trunk skeletogenesis, *Pax1* is also involved in formation of limb skeletal elements where it is initially expressed in the anterior proximal margin of limb buds (LeClair et al., 1999). Skeletal formation of the limb is subsequently orchestrated Sex determining region Y (SRY)-box 9/Sox9-positive mesenchymal cartilage progenitors that form condensations within the limb bud and undergo sequential endochondral ossification to form bone (Pineault and Wellik, 2014). As previously mentioned, limb skeletal muscle originates from cells within the somites, which then migrate to the developing limb bud (Cossu et al., 1996; Musumeci et al., 2015). These progenitor cells delaminate from the dorso-lateral region of the developing dermomyotome, become transiently inhibited from differentiating by expression of *Pax3* (Relaix et al., 2005), and subsequently express Tyrosine protein kinase met (*C-Met*; Cossu et al., 1996), a Hepatocyte growth factor (HGF) receptor. When *HGF* is expressed by mesenchymal cells of the limb bud, these muscle progenitors migrate toward these regions (Musumeci et al., 2015) and subsequently differentiate into muscle (Chevallier et al., 1977). Tendon progenitors arise from the lateral plate mesoderm and its specification is independent of muscle (Kieny and Chevallier, 1979). These tendon progenitors express *Scx*, and its protein levels are negatively- and positively-regulated by bone morphogenetic protein (BMP) and Noggin signaling, respectively (Schweitzer et al., 2001). Further musculoskeletal development of these tissues is interdependent. For example, removal of tendon primordia disrupts limb muscle formation (Kardon, 1998), whereas loss of limb muscle only results in transient tendon formation (Kieny and Chevallier, 1979). Other studies have also shown that the tendon/ligament marker *Scx* is crucial for musculoskeletal integration (Yoshimoto et al., 2017). Indeed, a population of cells positive for both *Scx* and *Sox9* are found at the interface between tendon and as-yet unmineralized bone, which contributes to eventual formation of the bone-tendon interface (Blitz et al., 2013; Sugimoto et al., 2013). For example, *Scx* has been shown to regulate *Bmp4* expression in tendon cells and this in turn subsequently directs formation of bone ridges at the deltoid tuberosity of humeral bone, which provides a stable anchoring point and stress dissipation for musculoskeletal tissue attachment (Blitz et al., 2009).

Thus, bone and tendon formation in the limb is derived from lateral plate mesoderm, whereas muscle formation originates from migrating precursors of paraxial mesoderm-derived somite.

### Role of Mechanical Forces in Musculoskeletal Tissue Maturation

**Figure 2:**
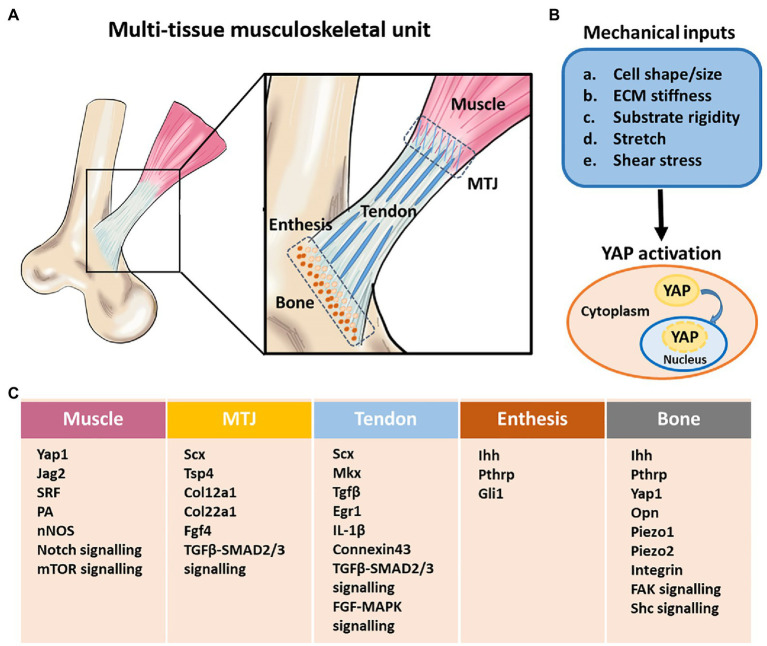
Structural components, and mechanobiological response and gene regulation in the multi-tissue musculoskeletal unit. **(A)** A schematic illustration of multi-tissue musculoskeletal unit containing bone, tendon, muscle, bone-tendon enthesis, and myotendinous junction (MTJ). The proper formation of tendon, muscle, and the attachment between them requires mechanical load. **(B)** Different mechanical inputs regulate Yes-associated protein (YAP) activity. YAP is localized to the nucleus and active under mechanical conditions that lead to high intracellular tension. **(C)** Mechanically regulated key signaling pathways, factors and genes involved in musculoskeletal system development. MTJ, myotendinous junction. Adapted with permission from Felsenthal and Zelzer (2017).

In addition to the specification, development, and integration of bone, tendon, and muscle, maturation of these musculoskeletal tissues occurs both *in utero* and postnatally. For obvious reasons, mechanical forces play a substantial role in muscle, tendon, and bone maturation. While this topic is broad, excellent reviews of this subject include those by Felsenthal and Zelzer (Felsenthal and Zelzer, 2017; mechanical forces during musculoskeletal development), Mammoto et al. (Mammoto et al., 2012), Jansen et al. (Jansen et al., 2015; mechanosensitive mechanisms), Geoghegan et al. (Geoghegan et al., 2019; bone mechanobiology), and Lavagnino et al. (Lavagnino et al., 2015; tendon mechanobiology), as well as Fischer et al. (Fischer et al., 2016) and Schiaffino et al. (Schiaffino et al., 2013; muscle mechanobiology; Figure 2).

One area of rapid advancement is the Hippo network, a highly conserved pathway associated with organ size regulation, mediated in part, by two transcriptional co-activators – Yes-associated protein (YAP) and Transcriptional co-activator with PDZ-binding motif (TAZ). YAP/TAZ activity is defined by its intracellular locations – nuclear YAP/TAZ is active as exemplified by culturing cells on a stiff substrate, which often results in a spread cell morphology; cytoplasmic YAP/TAZ is inactive as exemplified by culturing cells on a soft substrate, which often results in a rounded cell morphology. At the cellular level, musculoskeletal actors including osteoblasts, osteocytes, tenocytes, myocytes, and various progenitors are highly sensitive to mechanical forces, contributing toward the overall and final properties of the tissue. However, details of the relevant intracellular signaling mechanisms that transduce mechanical cues remain largely unclear. Recent studies have shown that YAP and TAZ are regulated by ECM elasticity and cell geometry (Dupont et al., 2011; Wada et al., 2011). Crucially, the control of YAP/TAZ subcellular localization by mechanical cues is highly conserved among different cell types such as adult tissue-derived mesenchymal stem cells (MSCs), which contribute toward musculoskeletal differentiation and tissue homeostasis. Indeed, the shifting of MSC differentiation from osteogenic (rigid substrate) to adipogenic (soft substrate) lineage is governed by YAP/TAZ activity (Dupont et al., 2011; Seo et al., 2013). Also, there is increasing evidence that YAP/TAZ signaling is implicated in multiple events of the musculoskeletal system, including the regulation of endochondral bone ossification and fracture healing (Deng et al., 2016; Li et al., 2018), articular cartilage maintenance (Deng et al., 2018b), collagen secretion from tendon fibroblasts (Yeung et al., 2015), and promotion of muscle growth (Fischer et al., 2016; Huraskin et al., 2016; Stearns-Reider et al., 2017). Future investigations are expected to elucidate how YAP/TAZ regulates and transduces various mechanical stimuli into intracellular signaling in these musculoskeletal cell types.

Like their tissue counterparts, the interfaces of musculoskeletal tissues are mechanosensitive and remain highly dynamic throughout life. For example, bone-tendon/bone-ligament interfaces, such as the medial collateral ligament and periodontal ligament, migrate with skeletal growth (Wang et al., 2013, 2014) and biomechanical loading (Lin et al., 2017), respectively, while the architecture and composition of muscle-tendon interfaces are highly responsive to exercise-induced loading (Jakobsen et al., 2017).

At the bone-tendon/bone-ligament interface or enthesis, four classically-distinct regions are defined – bone, mineralized fibrocartilage, unmineralized fibrocartilage, and tendon regions (Yang and Temenoff, 2009). Recent work has shown a greater degree of nuance, with a gradual transition in both mineral accumulation (Moffat et al., 2008; Genin et al., 2009; Schwartz et al., 2012) and organization of collagen fibers (Genin et al., 2009; Rossetti et al., 2017) observed across this interface. Together, these interfacial features not only mediate attachment of flexible tendon to rigid bone but also act to minimize the risk of tissue rupture and detachment by reducing stress concentrations. Growth factors and ECM components associated with the development and maturation of the humeral bone-supraspinatus tendon interface include TGF-β s, such as TGF-β1 and TGF-β3, as well as various fibrous collagen types, such as collagen types I, II, and X (Galatz et al., 2007).

Postnatal development of this interface is initiated at the tendon-epiphyseal cartilage/unmineralized bone interface by Hedgehog-responsive cells expressing the transcription factor Gli1 (Schwartz et al., 2015). For some entheses, these cells are synonymous with a *Scx*- and *Sox9*-positive embryonic cell population identified in prior studies (Blitz et al., 2013; Sugimoto et al., 2013; Felsenthal et al., 2018), and eventually divide into two subpopulations – one of which contributes toward formation of mineralized fibrocartilage and eventually becomes non-responsive to Hedgehog signaling, while the other contributes toward formation of unmineralized fibrocartilage and retains its responsiveness to Hedgehog signaling (Schwartz et al., 2015).

Muscle loading plays a crucial role in enthesis development, as botulinum toxin-induced muscle paralysis can alter Hedgehog signaling and disrupt enthesis maturation as well as biomechanical properties (Derwin et al., 2006; Deymier et al., 2019). Muscle unloading for 21 days affected both structure and biomechanical properties of musculoskeletal tissue units at multiple hierarchical levels (Deymier et al., 2019). At the macro scale, supraspinatus tendon tensile strength and bone-tendon interfacial toughness were decreased by 36.6–66.7% and 70.0–74.3%, respectively, whereas ultimate load and stiffness remained unaffected in muscle-unloaded samples (Deymier et al., 2019). At the nanometer scale, the level of bioapatite carbonate across the unloaded bone-tendon interface was decreased by 6.25%, which when coupled with changes in bioapatite crystal orientation led to decreased total energy dissipation (Deymier et al., 2019). As such, the resulting loss of organization in unloaded musculoskeletal tissue units across multiple length scales increased injury risk *via* a decreased ability to absorb energy prior to material failure (Deymier et al., 2019).

When fully-formed, bone-tendon/bone-ligament interfaces may be “stationary,” whereas others such as those found in the medial collateral ligament migrate with skeletal growth (Wang et al., 2013, 2014). During this process, load-induced Parathyroid hormone-related protein (PTHRP) causes bone resorption above the interface to form an unmineralized tract (Wang et al., 2013, 2014). This unmineralized region specifies the future destination of the migrating interface, and migration to this site occurs as a result of coupled osteoclast- and osteoblast-driven bone resorption and formation, respectively (Wang et al., 2013, 2014). Together, these studies highlight the dynamic nature of bone-tendon/bone-ligament interface.

At the muscle-tendon interface, three distinct regions, tendon, myotendinous junction (MTJ), and muscle, are formed (Yang and Temenoff, 2009). Together, these interfacial features not only mediate attachment of flexible tendon to the contractile force generation apparatus in skeletal muscle but also act to minimize the risk of tissue rupture and detachment by reducing stress concentrations.

Unlike the bone-tendon interface, these distinct regions are already formed during embryonic development and have already undergone *in utero* maturation to some extent. For example, the myotendinous interface of zebrafish embryos remodels from an ECM milieu that is originally rich in fibronectin to one that is laminin-rich (Jenkins et al., 2016). Laminins are crucial in this respect as they play important roles in both skeletal muscle integrity and force transmission (Holmberg and Durbeej, 2013). This is important as *in utero* muscle loading, such as kicking movements, are vital to regulation of musculoskeletal tissue mechanical properties, including tendon (Pan et al., 2018).

During postnatal maturation of the muscle-tendon interface, an increase in tissue complexity is observed (Nagano et al., 1998). For example, the ends of skeletal muscle fibers in Chinese hamsters undergo morphogenesis from relatively simple cone-shaped structures into complex structures with an increased number of finger-like projections and clefts. Such dynamic changes are likely a response to increased muscle loading and are echoed in studies, where different exercise regimens result in the remodeling of muscle-tendon organization and structure (Curzi et al., 2016; Rissatto Sierra et al., 2018).

In summary, both embryonic and post-natal development contribute toward the specification, maturation, and integration of bone-tendon-muscle units. Embryonic processes are primarily responsible for initiating cell differentiation and early multi-tissue patterning, whereas post-natal processes, under the influence of biomechanical loading, guide and regulate the maturation and remodeling of musculoskeletal interfaces.

## Musculoskeletal Tissue Healing and Graft Integration

### General Overview of Wound Healing

To effectively integrate engineered musculoskeletal grafts, it is important to understand the role of wound healing. In general, wound healing is comprised of four sequential but overlapping stages – hemostasis, inflammation, proliferation, and maturation (Marieb, 1999).

Briefly, wound healing first commences with hemostasis, which initiates inflammation, recruits cells, and mediates wound closure. During this phase, platelet activation and degranulation results in: (1) the initiation of coagulation *via* increased expression of cell surface protein αIIb/β3 (which exhibits high affinity for fibrinogen to form the provisional ECM) as well as the secretion of adenosine diphosphate (ADP) and von Willebrand factor, which aid in platelet adhesion and aggregation; (2) the release of a multitude of growth factors that orchestrate inflammation and recruit cells including but not limited to fibroblasts, immune cells such as macrophages, and stem cells, which participate in wound healing and regeneration (Marieb, 1999; Aurora and Olson, 2014; Eming et al., 2014; Manning et al., 2014). Second, inflammation establishes the initial wound microenvironment by removing both pathogens and damaged cells (Marieb, 1999; Aurora and Olson, 2014; Eming et al., 2014; Manning et al., 2014), as well as *via* the secretion of inflammatory mediators that attract stem and progenitor cells for subsequent tissue repair (Rustad and Gurtner, 2012). Third, proliferation regenerates tissue-resident cells *via* granulation tissue formation and myofibroblast-mediated wound contraction (Marieb, 1999; Aurora and Olson, 2014; Eming et al., 2014; Manning et al., 2014). Fourth, tissue remodeling removes transient cells and ECM, reorganizing and closing the wound to re-establish native tissue (Marieb, 1999; Aurora and Olson, 2014; Eming et al., 2014; Manning et al., 2014). However, if the injury is severe or chronic, such as in the case of tendinopathy, healing may be incomplete with the formation of mechanically-weaker scar tissues as well as the persistence of aberrant inflammatory conditions (Marieb, 1999; Aurora and Olson, 2014; Eming et al., 2014; Manning et al., 2014). Thus, wound healing is a crucial consideration for simultaneous engineering and integration of multiple musculoskeletal tissue grafts.

### Importance of Inflammation in Tendon, Skeletal Muscle, and Bone Healing

Given its aforementioned role in establishing the initial wound microenvironment, inflammation has emerged as a crucial consideration for musculoskeletal regeneration. For example, inflammatory cells such as macrophages were increased at the muscle-tendon interface following exercise (Jakobsen et al., 2017). Presumably, these macrophages are responsible for orchestrating tissue repair and adaptation as part of the body’s homeostatic program. Indeed, macrophages have been reported to organize the wound microenvironment for regenerating entire limbs in model organisms such as the adult salamander (Godwin et al., 2013).

With respect to tendon injury, many inflammation-associated mediators and cell types have been identified. These include Interleukins (ILs; IL-1β, IL-6, IL-10, IL-17A, IL-21, and IL-33), Tumor necrosis factor-alpha (TNF-α), Substance P, and alarmin molecules, as well as neutrophils, macrophages, mast cells, lymphocytes, fibroblasts, tenocytes, and stem/progenitor cells (Jensen et al., 2018; Tang et al., 2018; Vinhas et al., 2018). While the precise roles and contributions of these signaling molecules and immunocytes remain unclear and are actively being studied (Tang et al., 2018), it is evident that an unbalanced inflammatory response is detrimental, and is associated with rotator cuff overuse injuries (Perry et al., 2005) and tears (Yadav et al., 2009). It is also worth noting that the majority of rotator cuff tears involve tendinopathic changes. For example, pro-inflammatory-associated IL-1β treatment has been shown to increase expression of ECM-destructive enzymes including matrix metalloproteinases (MMPs) such as MMP-1, MMP-3, MMP-13 as well as aggrecanase-1 in human tenocytes (Tsuzaki et al., 2003), which positively correlate with increased rotator cuff tear size and severity (Shindle et al., 2011). However, this does not imply that direct inhibition of pro-inflammatory responses will thus lead to improved tendon healing. For example, non-steroidal anti-inflammatory drugs, including indomethacin and celecoxib, are often prescribed after orthopaedic procedures to alleviate pain. In an acute rat rotator cuff injury study, depressing the inflammatory response using indomethacin or celecoxib also retarded tendon healing with decreased collagen organization and inferior biomechanical properties (Cohen et al., 2006).

**Figure 3:**
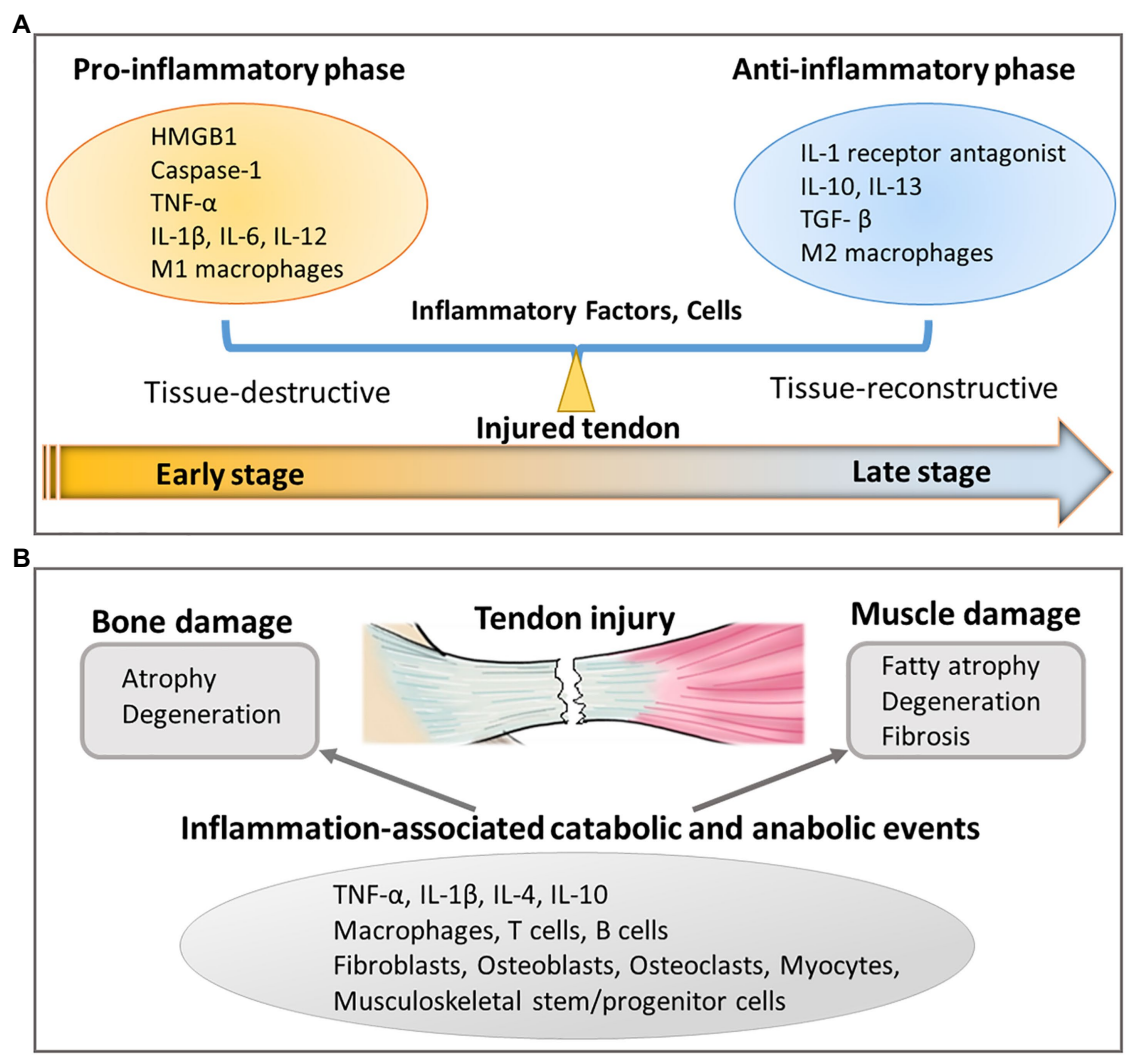
Inflammation-related responses resulting from tendon injury. **(A)** Unbalanced pro-inflammatory and anti-inflammatory response in injured tendon affects tendon healing by secreting inflammatory factors and cells. **(B)** Tendon injury also leads to bone and muscle damage through inflammation-associated catabolic and anabolic events.

Both classically pro- and anti-inflammatory responses are necessary for tendon healing. Following tendon tissue damage, alarmin molecules such as High-mobility group box 1 (HMGB1) along with other pro-inflammatory molecules, including caspase-1, IL-1β, receptor for advanced glycation end products, Toll-like receptors (TLRs) such as TLR-2 and TLR-4, and triggering receptor expressed on myeloid cells were upregulated at early time points (1–2 weeks) and subsequently declined (3–4 weeks), which correlated well with the healing response (Thankam et al., 2018). Although, the secretion of pro-inflammatory molecules such as IL-1β is associated with ECM-degradation, such effects are presumed to be beneficial for tendon remodeling and repair when induced at moderate levels (Yang et al., 2005). Stromal cell-derived factor-1, an associated downstream mediator of pro-inflammatory IL-1β (Akbar et al., 2017), then mediates the next phase of wound healing by facilitating infiltration of pro-regenerative M2 macrophages and bone marrow-derived stem cells into supraspinatus muscle (Tellier et al., 2018). At later stages of tendon healing, anti-inflammatory-associated IL-10 is expressed (Ricchetti et al., 2008; Sugg et al., 2014) and promotes proliferation and migration of tendon-derived stem cells concurrently with inhibition of tenocyte differentiation (Deng et al., 2018a). Taken together, these studies demonstrate that a delicate balance between tissue-destructive pro-inflammatory and tissue-reconstructive anti-inflammatory responses is crucial for chemotaxis and expansion of musculoskeletal stem/progenitor cells into physiologically relevant numbers sufficient for effecting tendon modeling and repair (Figure 3). When this delicate balance is disrupted, such as in the case of persistent inflammation, chronic ECM degradation leads to disorganized tendon ECM organization and results in mechanically weak scar tissue formation.

Similar to tendon, inflammation also has catabolic and anabolic effects on skeletal muscle and bone. An over-exuberant pro-inflammatory response induces bone resorption (Goldring, 2015) and muscle wasting (Londhe and Guttridge, 2015), which are linked to the pathophysiology of joint diseases, including rheumatoid arthritis and seronegative spondyloarthropathies (Goldring, 2015; Londhe and Guttridge, 2015). Similar inflammatory mediators, including TNF-α, IL-1β, IL-4, and IL-10, as well as cell types, including macrophages, T cells, B cells, fibroblasts, osteoblasts, osteoclasts, myocytes, and musculoskeletal stem/progenitor cells, are involved (Goldring, 2015; Londhe and Guttridge, 2015; Turner et al., 2018; Figure 3).

As such, inflammation-associated catabolic and anabolic events can affect patients afflicted by musculoskeletal disease or injury. In rheumatoid arthritis, joint-resident synovial fibroblasts, by virtue of their immune-associated cell receptors and chemokine secretion, can both respond to and promote inflammation (Turner et al., 2018). This response allows them to modulate leukocyte infiltration as well as the activity of bone-building osteoblasts and bone-resorbing osteoclasts, respectively, to perturb bone remodeling (Turner et al., 2018). This is important in the context of rotator cuff repair, since the number of shoulder arthroplasty patients with rheumatoid arthritis is increasing (Leroux et al., 2018), and increased rotator cuff tear severity is positively correlated with increased synovium inflammation (Shindle et al., 2011). Similarly, in rotator cuff muscles, increased macrophage density (10–100-fold relative to healthy tissue) has been observed (Gibbons et al., 2017). Presumably, these increased macrophage numbers contribute toward fatty atrophy and fibrosis of skeletal muscle tissue *via* Ras homolog gene family member A (RhoA) signaling (Davies et al., 2017). Rebalancing pro- and anti-inflammatory responses *via* application of anti-inflammatory drugs such as Licofelone (Oak et al., 2014) and Simvastatin (Davis et al., 2015) decreased muscle fibrosis. Thus, a crucial balance between pro- and anti-inflammatory is also necessary to prevent atrophy/degeneration/retraction of skeletal muscle and bone components of a musculoskeletal unit.

### Importance of Inflammation in Integration of Clinical Grafts and Devices

In addition to their anabolic and catabolic effects on musculoskeletal tissues, inflammation also plays a pivotal role in considering the efficacy of medical devices such as sutures, suture anchors, and grafts. Such inflammation-associated responses share similarities with but are ultimately distinct from those involved in disease progression or normal wound healing. For example, in an acute rabbit supraspinatus tendon resection model, non-absorbable sutures induced higher levels of inflammation relative to absorbable and partially-absorbable sutures (Su et al., 2018). This increased pro-inflammatory response was associated with poorer biomechanical properties and incomplete bone-tendon healing (Su et al., 2018). Similarly, severe pro-inflammatory responses associated with either tendon grafts (Walton et al., 2007) or suture anchors (Barber, 2007; Cobaleda Aristizabal et al., 2014; Joo Han et al., 2015) have also led to failed rotator cuff repairs, requiring patients to undergo revision surgeries.

For these circumstances, it is challenging to ascertain a single underlying cause of failure. However, an important consideration is the non-autogenic nature of these clinical devices (Barber, 2007; Walton et al., 2007; Cobaleda Aristizabal et al., 2014; Joo Han et al., 2015; Su et al., 2018), which can induce different levels of inflammation. Indeed, xenogenic grafts induced an adverse pro-inflammatory response (Walton et al., 2007), whereas allogenic grafts had minimal levels of inflammation (Snyder et al., 2009). In addition, scaffolds and clinical grafts can also be broadly divided into biological, synthetic, or biosynthetic (hybrid of biological and synthetic) in origin. Typically, synthetic scaffolds are favored for their ease of production and reproducible attributes such as mechanical properties, which is important for facilitating joint locomotion, but have traditionally been considered as strong inducers of chronic inflammation and the foreign body response relative to their biological counterparts (Crupi et al., 2015; Saleh and Bryant, 2018; Zhou and Groth, 2018; Sadtler et al., 2019). Indeed, in a muscle wound environment, synthetic materials (polyethylene and polyethylene glycol) elicited a type-1-like immune response with recruitment of a high proportion of neutrophils and CD86-positive pro-inflammatory macrophages, which was attributed to biomaterial stiffness, whereas a biological material (porcine urinary bladder ECM), elicited a type-2-like immune response with upregulation of *Il4*, *Cd163*, *Mrc1*, and *Chil3* genes, which was attributed to damage-associated molecular patterns signaling (Sadtler et al., 2019). Similarly, in a bone wound environment, inflammatory mediators such as TLR-4 have been shown to play vital roles in mediating calvarial bone healing as well as bone graft-mediated calvarial repair (Wang et al., 2015, 2017).

The magnitude of inflammation and the ensuing foreign body reaction are highly dependent upon immune cell-biomaterial interactions which occur at the tissue/material interface (Anderson et al., 2008). Following implantation of a clinical device, plasma proteins are adsorbed, or opsonized onto the biomaterial surface followed by complement activation, macrophage adhesion, and macrophage fusion into foreign body giant cells that mediate degradation (Anderson et al., 2008). If this opsonization process creates a detrimental or chronic wound microenvironment, device failure and impaired shoulder function will result (Walton et al., 2007), whereas if a microenvironment conducive to regeneration is presented, graft remodeling and successful integration with host tissue will ensue (Snyder et al., 2009). Contributing factors related to this include biomaterial stiffness, biomaterial surface chemistry, scaffold/graft topography, scaffold/graft degradation rate, and physico-chemical effect of degradation products, scaffold/graft porosity, and presence of inflammatory antigens such as α-galactosidase (Crupi et al., 2015; Zhou and Groth, 2018; Sadtler et al., 2019). This has led to the development of diverse anti-inflammatory strategies, which include use of scaffold topography such as grooves to reduce immune cell pro-inflammatory response, modification of biomaterial surfaces to present weakly inflammatory functional chemical groups such as hydroxyls and carboxylic acids, use of anti-fouling coatings, attachment of anti-inflammatory or immunomodulatory reagents, and removal of inflammatory antigens such as α-galactosidase *via* enzymatic treatment (Crupi et al., 2015; Zhou and Groth, 2018). Thus, these studies highlight the importance of biomaterial or scaffold/graft characteristics and their impact on inflammation, which ultimately determines successful engraftment of devices used in clinical repair.

### Importance of Vascularization for Clinical Grafts and Devices

In tissue engineering, one major challenge for the long-term survival and function of clinical implants and devices is ensuring sufficient vascularization after implantation. There are two principal strategies are used – angiogenesis and inosculation. Angiogenesis focuses on stimulating the ingrowth of newly formed blood vessels into implanted constructs from the surrounding host tissue(s), whereas the inosculation requires a preformed microvascular networks generated within tissue constructs that subsequently develop interconnections with the host microvasculature (Laschke and Menger, 2012). Strategies to address this include modification of scaffolds/grafts to induce a mild proinflammatory response that may be beneficial to vascularization, incorporation of signaling molecules that promote angiogenesis or blood vessel maturation such as FGF-2, PDGF, VEGF, TGF-β, Angiopoietin-1, and Angiopoietin-2, and incorporation of vascular or proangiogenic cells. The details of the vascularization process as well as the principles and strategies are extensively described by Laschke and Menger (2012) and Herrmann et al. (2014).

In particular, angiogenesis can also be impacted by the magnitude of inflammation. In a recent study, mice were intraperitoneally administered with arthritic serum repeatedly, resulting in clinical manifestations of rheumatoid arthritis including prolonged systemic inflammation (Wang et al., 2021a). Following bone fracture injury, such mice exhibited fracture nonunion with reduced cartilaginous and bony callus formation, fibrotic scarring, and diminished angiogenesis (Wang et al., 2021a). Subsequent mechanistic studies showed that this reduced angiogenesis was attributed to downregulation of SPP1 and CXCL12 in chondrocytes (Wang et al., 2021a). By employing a biodegradable polycaprolactone scaffold loaded with pro-angiogenic SPP1 and CXCL12, improved angiogenesis and osteogenesis, as evidenced by increased blood vessel count, increased bone area and higher biomechanical properties (torque) were achieved (Wang et al., 2021a). In a similar vein, different scaffolds and grafts can present varying levels of angiogenesis and vascular ingrowth, which can be affected by the magnitude of inflammation. In a mouse subcutaneous implantation study, 3D-printed poly(L-lactide-co-glycolide) and 3D-printed collagen-chitosan-hydroxyapatite hydrogel scaffolds were directly compared (Rücker et al., 2006). Intravital fluorescence microscopy analyses, histological as well as immunohistochemical staining, and cytotoxicity assays showed that 3D-printed poly(L-lactide-co-glycolide) scaffolds induced a mild pro-inflammatory response (slight increase in leukocyte recruitment), which was associated with increased angiogenesis and good microvascular ingrowth 14 days post-implantation, whereas 3D-printed collagen-chitosan-hydroxyapatite hydrogel scaffolds induced a severe pro-inflammatory response (around 15-fold increase of leukocyte-endothelial cell interactions), which was associated with poor microvascular ingrowth to the scaffolds (Rücker et al., 2006). Thus, scaffold or graft neovascularization, which is crucial to the long-term survival and function of an implant is dependent on a moderate pro-inflammatory response.

In summary, both pro- and anti-inflammatory responses are essential for healing of musculoskeletal tissue units and contribute toward integration of medical devices including vascularization. Pro- and anti-inflammatory responses are primarily, although not exclusively, responsible for catabolic and anabolic tissue responses, respectively, and a coordinated as well as balanced inflammatory response is essential to successful biomaterial integration and tissue repair.

## Perspective: What can we Learn From Developmental Biology and Wound Healing for Musculoskeletal Tissue Engineering

### Applicability

Concepts and knowledge generated from developmental biology and wound healing studies can aid the development and assessment of musculoskeletal tissue engineering strategies. In other words, information gained from developmental biology can offer a “cheat sheet” for assessing post-natal musculoskeletal differentiation as well as inspire novel therapeutic strategies to regenerate and repair injured musculoskeletal tissues (Tuan, 2004; Huang et al., 2015). For bone, growth factors such as BMPs, Wnts, and Indian Hedgehog have been identified as bone-promoting cues, while collagen type I, Runt-related transcription factor 2 (RUNX2), BMP-2, and BMP-6 serve as useful ECM and transcription factor markers to assess osteoblast differentiation (Wang et al., 2020). For tendon, growth factors such as TGF-βs, FGF, BMPs, Wnts, and Connective tissue growth factor (CTGF) have been identified as tendon-promoting cues, while collagen types I and III, tenascin-C, TNMD, decorin, biglycan, SCX, mohawk (MKX), and Early growth response 1 (EGR1) serve as useful ECM and transcription factor markers to assess tenocyte differentiation (Huang, 2017; Liu et al., 2017). For skeletal muscle, growth factors such as insulin-like growth factor-1, HGF, FGF-2, and PDGF have been identified as skeletal muscle-promoting cues (Syverud et al., 2016), while collagen type I, laminins, myogenin, MYOD, MYF5 serve as useful ECM and transcription factor markers to assess myocyte differentiation (Holmberg and Durbeej, 2013; Syverud et al., 2016; Huang, 2017). In addition to identification of appropriate cues and markers, signaling pathways governing musculoskeletal tissue formation can be manipulated to develop novel therapies. For example, stable cartilage formation has been achieved using targeted chemical inhibition of the BMP pathway without affecting TGF-β signaling (Occhetta et al., 2018). Similarly, stable cartilage formation has also been observed *in vivo via* application of the Wnt/β-catenin inhibitor XAV939 to manipulate Wnt signaling, which is crucial in the development, growth, and maintenance of both cartilage and bone (Deng et al., 2019). These studies demonstrate a remarkable achievement as prior attempts at stable chondrocyte differentiation typically result in a transient chondrocyte phenotype that rapidly proceeds toward a hypertrophic and mineralizing fate, eventually forming unwanted bone (Correa et al., 2015). Thus, integrating knowledge gleaned from developmental biology studies into tissue engineering approaches can prove valuable.

Wound healing studies can model injury-relevant settings for understanding the pathophysiological process as well as evaluating novel therapeutic strategies. For understanding pathophysiological processes, various models have been utilized. In rats, the supraspinatus tendon passes under an enclosed arch similar to humans (Soslowsky et al., 1996). This similarity in shoulder anatomy makes the rat ideal for modeling rotator cuff diseases such as subacromial impingement, whereby friction between the rotator cuff tendons and the coracoacromial arch during muscular movement leads to pain and reduced range of joint motion. In rabbits, detachment of the rotator cuff tendon(s) resulted in prominent fatty degeneration of skeletal muscle (Abdou et al., 2019), and has been used to simulate chronic rotator cuff tears to study the effect of hypercholesterolemia on skeletal muscle fatty degeneration and bone-to-tendon healing. Indeed, use of such models identified αSMA-positive cells as primary contributors to scar formation (Moser et al., 2020), which may pave the way for identification of new therapeutic targets. For evaluating novel therapeutic strategies, similar animal models have been used. For example, both rats and rabbits have been used to evaluate the effect of growth factors, such as FGF-2, Platelet-derived growth factor-BB (PDGF), and TGF-β1, on musculoskeletal tissue healing for rotator cuff repair (Tokunaga et al., 2015a,b, 2017; Arimura et al., 2017). Also, when coupled with appropriate outcome measurements, animal models can be used to understand why therapeutics may result in unfavorable outcomes. For example, biomechanical, biochemical, and proteomics studies showed that application of adipose-derived stromal cells with tenogenic BMP-12 in a fibrin-based scaffold for intrasynovial tendon repair amplified unfavorable responses including inflammation, stress response, and matrix degradation, leading to poor healing (Gelberman et al., 2016). Such studies highlight the need for avoidance of negative local reactions in cell- and growth factor-based therapies.

Thus, knowledge derived from developmental biology and wound healing studies can guide both the development and assessment of therapies for musculoskeletal tissue engineering and regenerative medicine by identifying musculoskeletal regenerative cues, differentiation markers, and potential therapeutic targets as well as facilitate evaluation of such therapies in injury-relevant models.

### Limitations

Despite the tremendous advances and insights gleaned from developmental biology and wound healing studies, there are noteworthy limitations. Thus, great care must be taken in interpreting their results and integrating this information in translational medicine efforts for two reasons.

First, within the context of developmental studies, there are intrinsic differences between non-adult and adult musculoskeletal cells as well as their tissue environments. For example, it is widely known that adult tendons have incomplete healing, which entails scar tissue formation (Longo et al., 2008, 2011), whereas fetal or neonatal tendons exhibit scarless wound healing (Andarawis-Puri et al., 2015; Galatz et al., 2015; Howell et al., 2017). In a mouse Achilles tendon resection study, improved neonatal healing was attributed to recruitment of Scx-positive cells, which are absent in adults (Howell et al., 2017). In lieu of Scx-positive cells, smooth muscle α-actin-positive cells mediated wound healing *via* fibrovascular scar tissue formation, leading to impaired musculoskeletal function (Howell et al., 2017). Transplantation of wounded adult and fetal sheep tendons into immunocompromised adult mice demonstrated that fetal cells exhibited superior intrinsic healing capacities (Favata et al., 2006). Wounded fetal tendons demonstrated both wound closure and recovery of tendon biomechanical properties including peak stress, peak load, modulus, and stiffness relative to unwounded controls, while wounded adult tendons did not (Favata et al., 2006). Also, fetal or postnatal tissue-specific microenvironments differ from that in adult tissue and can alter the wound healing response. These differences include high cellularity and low ECM content in developing tendons, which stand in stark contrast to the hypocellular and predominantly ECM-based nature of adult tendons (Dahners, 2005). For example, subtle compositional differences in the ECM of developing tendons such as lysyl oxidase-mediated collagen crosslinking increased mechanical properties such as stiffness (Marturano et al., 2013), which can affect tenogenic differentiation (Schiele et al., 2013). Another major difference in tissue environment includes differences in the inflammatory microenvironment between non-adult and adult tendon (Galatz et al., 2015). To-date, direct comparisons between adult and fetal tendon inflammation have not been reported, but in related musculoskeletal systems such as articular cartilage, improved wound healing is typically associated with lower inflammatory response (Ribitsch et al., 2018). However, more studies are needed to identify potential inflammatory factors associated with improved wound healing (Galatz et al., 2015). This is because the relationship between cells and their tissue environments is highly dynamic and interdependent. For example, dermally-derived fetal fibroblasts exhibited greater regenerative potential relative to their adult counterparts (Tang et al., 2014). Using a 2 mm mouse Achilles tendon defect model, tendon cell sheets engineered from fetal cells demonstrated improved biomechanical properties, such as higher stiffness and tensile modulus, as well as tissue organization, such as increased tissue birefringence and collagen fibril size (Tang et al., 2014). Improved tendon healing in this scenario was attributed to increased fetal fibroblast proliferation and decreased immunocyte presence at the injury site concomitant with decreased expression of pro-inflammatory cytokines including IL-1β, IL-6, and CD44 (Tang et al., 2014). Such studies highlight the need for careful interpretation of findings from developmental studies for therapeutic development.

Second, knowledge gleaned from the study of animal models has limitations. Developmental and wound healing studies have used a vast array of model organisms to advance our understanding of tendon biology. These include invertebrates such as drosophila (Schweitzer et al., 2010), vertebrates such as fish (Chen and Galloway, 2014, 2017), chick (Schweitzer et al., 2001; Edom-Vovard et al., 2002; Edom-Vovard and Duprez, 2004; Havis et al., 2016), and mice (Thomopoulos et al., 2007; Schweitzer et al., 2010; Schwartz et al., 2012, 2013, 2015, 2017; Huang et al., 2015; Havis et al., 2016; Huang, 2017). However, species-specific differences may limit the applicability of growth factors identified for musculoskeletal tissue engineering. For example, it has been suggested that FGF signaling is important for tendon development in chick but less so for mouse (Havis et al., 2016). Also, TGF-β3 is implicated in tendon development (Liu et al., 2017) but its application resulted in more disorganized scar tissue relative to repair only and carrier only controls (Manning et al., 2011). In addition, there is growing recognition that the hierarchical nature of tendon organization in smaller animal models such as rats, while useful for studying musculoskeletal biology, do not mimic those seen in larger organisms, such as horse or humans, owing to their lack of high load-bearing fascicles (Lee and Elliott, 2018). Also, it is imperative to both devise an appropriately relevant injury model for evaluation. For example, severe rotator cuff tears are typically chronic in nature (Gartsman, 2009). As such, chronic injury models, such as those which entail skeletal muscle fatty degeneration (Gupta and Lee, 2007), are more ideal for evaluating a therapy meant to address large-to-massive injuries. At the same time, it is important to carefully interpret animal model studies that employ acute injuries for an intervention meant to address a chronic wound. Such studies highlight the need for careful design of studies and interpretation of their results.

Thus, developmental biology and wound healing studies can offer tremendous insights that aid musculoskeletal tissue engineering and regenerative medicine, but factors such as intrinsic differences between non-adult and adult cells and microenvironment, species-specific differences, and relevance of wound model must be carefully considered.

## Outlook

In summary, developmental and wound healing studies can serve as useful inspirations to engineer multi-tissue, musculoskeletal units. Most importantly, it is evident that musculoskeletal development and integration of bone, tendon, and muscle tissues are highly interdependent, while common inflammatory and anti-inflammatory signals mediate healing of these tissues as well as integration of clinical grafts. Collectively, such inquiries have advanced our understanding of musculoskeletal biology by identifying useful markers of musculoskeletal differentiation and promising cues for therapeutic development. However, integrating this knowledge for translational applications requires careful consideration of differences in embryonic or postnatal development with adult wound healing as well as the limitations of animal models used for studying developmental biology and simulating injury. Overcoming such differences will greatly advance efforts to engineer or regenerate multi-tissue musculoskeletal units.

## Author Contributions

XZ wrote the manuscript and prepared the figures. DW and K-LM wrote the manuscript. DK and RT conceived and wrote the manuscript. All authors contributed to the article and approved the submitted version.

## Conflict of Interest

The authors declare that the research was conducted in the absence of any commercial or financial relationships that could be construed as a potential conflict of interest.

## Publisher’s Note

All claims expressed in this article are solely those of the authors and do not necessarily represent those of their affiliated organizations, or those of the publisher, the editors and the reviewers. Any product that may be evaluated in this article, or claim that may be made by its manufacturer, is not guaranteed or endorsed by the publisher.

## Figures and Tables

**Table 1:** 

Terms	Definitions
ADP	Adenosine diphosphate
BMP	Bone morphogenetic protein
CTGF	Connective tissue growth factor
EGR1	Early growth response 1
ECM	Extracellular matrix
FGF	Fibroblast growth factor
HGF	Hepatocyte growth factor
HMGB1	High-mobility group box 1
HOX	Homeobox
IL	Interleukin
MMP	Matrix metalloproteinase
MKX	Mohawk
MYOD	Myogenic differentiation 1
MYF5	Myogenic factor-5
PTHRP	Parathyroid hormone-related protein
PAX	Paired box protein
PDGF	Platelet-derived growth factor
RHOA	Ras homolog gene family member A
RUNX2	Runt-related transcription factor 2
SCX	Scleraxis
SHH	Sonic hedgehog
SOX9	SRY (Sex determining region Y)-box 9
TNMD	Tenomodulin
TGF	Transforming growth factor
TAZ	Transcriptional co-activator with PDZ-binding motif
TLR	Toll-like receptor
TNF-α	Tumor necrosis factor-alpha
C-Met	Tyrosine protein kinase met
VEGF	Vascular endothelial growth factor
WNT	Wingless/integrated
YAP	Yes-associated protein
